# Response to “comments on: involving service users in the qualitative analysis of patient narratives to support healthcare quality improvement

**DOI:** 10.1186/s40900-019-0158-y

**Published:** 2019-09-11

**Authors:** Louise Locock, Susan Kirkpatrick, Lucy Brading, Gordon Sturmey, Jocelyn Cornwell, Neil Churchill, Glenn Robert

**Affiliations:** 1 Health Services Research Unit, University of Aberdeen, Health Sciences Building, Foresterhill, Aberdeen, AB25 2ZD UK; 2 Nuffield Department of Primary Care Health Sciences, University of Oxford, Woodstock Rd, Oxford, OX2 6GG UK; 3 Institute of Psychology Health and Society/North West Hub for Trials Methodology Research, University of Liverpool, Liverpool, L69 3GL UK; 4 Aberdeen, UK; 5 Point of Care Foundation, 99 Gray’s Inn Rd, London, WC1X 8TY UK; 6 Participation and Equalities, NHS England, Quarry House, Quarry Hill, Leeds, LS2 7UE UK; 7 Florence Nightingale Faculty of Nursing, Midwifery and Palliative Care, King’s College London, James Clerk Maxwell Building, 57 Waterloo Rd, London, SE1 8WA UK

Thank you for commenting on our paper [1]. Firstly, we agree that it is possible for people to engage with ‘raw data’; the ‘gold standard’ approach referred to by Jennings et al. [2] involves people engaging deeply with transcripts, to “achieve meaning at a deep, semantic level, and undertake extensive co-revision of themes, codes and frameworks as findings emerge. In essence, if the academic researchers are doing it, so are the PPI co-researchers.”

It is worth noting that in our case we were conducting a secondary analysis of existing data collected some time ago. While we had PPI partners involved throughout our secondary analysis project, by definition that group of people could not have been involved in the data collection. Thus we were looking for ways to involve people in exploring this previously collected data from a new angle. Nonetheless, as we noted in our paper, some of the people who got involved with our project were really enthusiastic about engaging directly with transcripts. It is good to hear of your positive experience of reading and engaging with selected transcripts, and we agree that this may be easier when a group of people have been involved in primary data collection, a point also made by Jennings.

However, we would also argue that it is important not to let the best be the enemy of the good. Sometimes there may be more pragmatic ways to approach user involvement in analysis which make good use of people’s time and insights, and potentially bring a wider range of perspectives to bear. If we involve only those people willing to read full transcripts we could miss important insights from a wider group of people who might like to contribute in a different way. A full qualitative research dataset – say 40 interviews of an hour and a half each – can easily run to 1500 pages of text. Even reading 1500 pages, let alone coding the content and relating it to previous literature and theory, is a substantial undertaking (and one which, incidentally, is often under-estimated by those unfamiliar with qualitative research). The idea that an analytic conversation might be a helpful alternative approach was developed with and by our PPI partners, including two PPI co-authors, and is not simply a researcher view. Offering individuals a choice of ways to inform analysis can be helpful for them and enhance diversity.

In your group, using Garfield’s approach [3], you each read a few selected transcripts, and then brought your insights to a discussion of themes with the researcher. This sounds very much like an analytic conversation, but with a starting point in individual readings rather than a group discussion of likely anticipated themes. This is of course another good way to stimulate analytic conversation, rooted in raw data but not expecting people to analyse a whole dataset. In fact one of us is doing something similar in a current project where there was PPI involvement from the beginning in shaping the proposal and the interview guide, and now towards the end we are sharing selected transcripts and inviting PPI reflections on the emerging themes which will inform the researcher’s coding and analysis.

We would note that we did not ask people to share with us their experiences as such; we asked them to propose touchpoints drawing on or informed by their own experience. These touchpoints were intended to be generic likely issues for us to look out for (such as ‘noise’ or ‘support after discharge’), and where service improvement efforts could be focussed. We think this bringing of personal experiential insights (in this case to inform analysis and the identification of improvement priorities) is part of the point of PPI, but that is very different from treating it as data.

There are different ways to approach user involvement in qualitative data analysis, depending on the nature of the topic, the type of project (e.g. secondary versus primary analysis), how applied or theoretical the study is, and the skills, preferences and interests of the people involved. As Jennings et al. note, limited time and financial/human resources are another factor, and in “most funded studies….compromises between quality and pragmatism are required”. We would suggest there is no one “right” way, but a plurality of good ways, and we all learn by openly sharing our experiences, our successes and our challenges.

## Data Availability

Not applicable.
